# Powerful yet Disempowered: A Thematic Literature Review Exploring the Challenges of Media Reporting on Sexual Violence

**DOI:** 10.1177/01968599251348248

**Published:** 2025-06-11

**Authors:** Karen Andrews, Safeera Jaffer, Shaheen Shariff

**Affiliations:** Department of Integrated Studies in Education, McGill University, Montreal, Canada

**Keywords:** sexual violence, journalism education, media reporting, ethical guidelines, training, #MeToo

## Abstract

Since #MeToo (2017), media discourse has brought sexual violence into greater public consciousness. Despite certain gains in how journalists frame stories of sexual violence, issues such as rape myths and victim blaming continue in reporting practices. This thematic literature review identified 41 articles on sexual violence reporting practices from the Global North since 2013. Seven themes emerged, including five related to the content of media reporting and two related to the process: (1) prevalence of rape myths and rape culture, (2) language of blame, (3) problematic media framing, (4) ignored intersectionality, (5) biased use of sources, (6) structural challenges for journalists, and (7) lack of education, training, and practical engagement with ethical guidelines. The literature demonstrates significant gaps post #MeToo in ethical reporting on sexual violence because issues are contextually entrenched in systems of oppression, and much more work must be done to resist prominent stigma and stereotypes.

Media discourse has brought sexual violence into greater public view, and traditional print and digital media wield immense power through news stories (Bohner, 2001; Clark, 1992; Hewa, 2021; Starkey et al., 2019; Su et al., 2022). Despite gains in media framing (Aroustamian, 2020), sexual violence reporting continues to perpetuate rape myths following #MeToo (2017) (Sacks et al., 2018; Sampert, 2010). This matters because media serve as proxy public educators on issues including sexual violence (Hindes & Fileborn, 2019). Just as news media educate the public, scholarly research facilitates knowledge exchange, enabling in-depth analyses of sexual violence. The goal of this paper is to examine the tensions between advances and constraints of contemporary media reporting, training, and the structural challenges journalists face when covering sexual violence.

Over 30% of women and girls in Canada over 15 have experienced sexual assault, along with 13% of men (Khan et al., 2023), excluding the higher rates of sexual violence committed against 2SLGBTQI + (Two-Spirit, Lesbian, Gay, Bisexual, Trans, Queer, Intersex, +) people (Cobb & Horeck, 2018), Indigenous people, and racialized and minoritized women (Cripps, 2021). Up to 95% of survivors do not report their assaults (Cotter & Savage, 2019; Sampert, 2010; Shariff & Eltis, 2017) and nearly 20% of sexual assaults reported to police are closed before reaching the legal system (Doolittle, 2017). Reports are often dismissed due to policing structures rooted in patriarchy and oppression (Braganza, 2020; Maynard, 2017; Puddister & McNabb, 2024). Sexual violence and gender-based violence are often framed as private violence, but private and public violence are not binary opposites; they exist on a continuum of violence that affects everyone (Hindes & Fileborn, 2019; Mass Casualty Commission, 2023). Kelly's (1988) continuum of violence theory situates violence within everyday context and experiences rather than fixed, episodic, or specifically defined by legal parameters. This framework guides our analysis, emphasizing that increased media coverage alone does not guarantee improved reporting (Cuklanz, 2020).

The original #MeToo movement, initiated by Tarana Burke in 2006 supporting Black sexual violence survivors, gained widespread attention in 2017 with allegations against Harvey Weinstein and other high-profile perpetrators (Cuklanz, 2020). #MeToo forced the news media to focus more on survivors’ stories, which signaled a breakthrough in media reporting conventions (Starkey et al., 2019). Although #MeToo (2017) has been criticized for prioritizing white, cisgender women's experiences (Su et al., 2022), it influenced media framing of sexual violence (Evans, 2018; Starkey et al., 2019). While the movement led to increased digital coverage, it also amplified online hostility to survivors on social media (Braganza, 2020). The normalization of violence is partly reinforced by “manfluencers” who spread misogynistic, male, and often white-dominated content, upholding rape culture and myths post #MeToo (Vail, 2023). These historical shifts continue to shape journalism and media reporting.

Recognizing the power of language, we define key terms in this literature review. Khan et al. (2023) describe sexual violence as “abusive and violent behaviours including, but not limited to, rape, sexual harassment, molestation, unwanted sexual contact, stalking, and voyeurism” (p. 11). While our focus is on sexual violence, we acknowledge its place within broader gender-based violence. We use the term “survivor” over “victim” or “victim-survivor,” except in direct quotations (Khan et al., 2023). Though personal terminology varies and acronyms can be reductive, we use the term 2SLGBTQI + .

This study is part of a larger research project on sexual violence in universities and society. For this study, we conducted a thematic literature review on advances and constraints in contemporary media reporting and training related to sexual violence news coverage. Rather than looking at news media texts directly, we examined scholarly findings on reporting practices. This approach identifies key contemporary themes in sexual violence reporting, demonstrates its prevalence in the Global North, and links journalistic practices to structural issues.

Our main research question is: what does academic literature reveal about news media reporting on sexual violence pre- and post-2017 #MeToo movement in the Global North? The two sub-questions are: what are the intricate issues of journalism reporting on sexual violence within the last 10 years, particularly related to rape culture, language, and framing? What structural and educational barriers do journalists face in ethically reporting on sexual violence?

## Methods

### Literature Parameters

To conduct this literature review, we began with the key concepts of education, journalism, and sexual violence reporting. We set specific parameters based on the research questions, focusing our search on articles relevant to journalism on sexual violence, ethical reporting practices on trauma, and education and training. While important research on media reporting of sexual violence exists globally, particularly in South Asia (Bamezai et al., 2020), the scope was narrowed to focus on the Global North, including Canada, the United States, Australia, New Zealand, and Europe. We considered articles published between 2013 and 2023 to prioritize the contemporary role of news media and social media. Considering the delay in academic publishing, this 10-year timeframe allowed us to take a comparative approach before and after the 2017 #MeToo movement.

### Searching for and Identifying Relevant Articles

**Figure 1.**
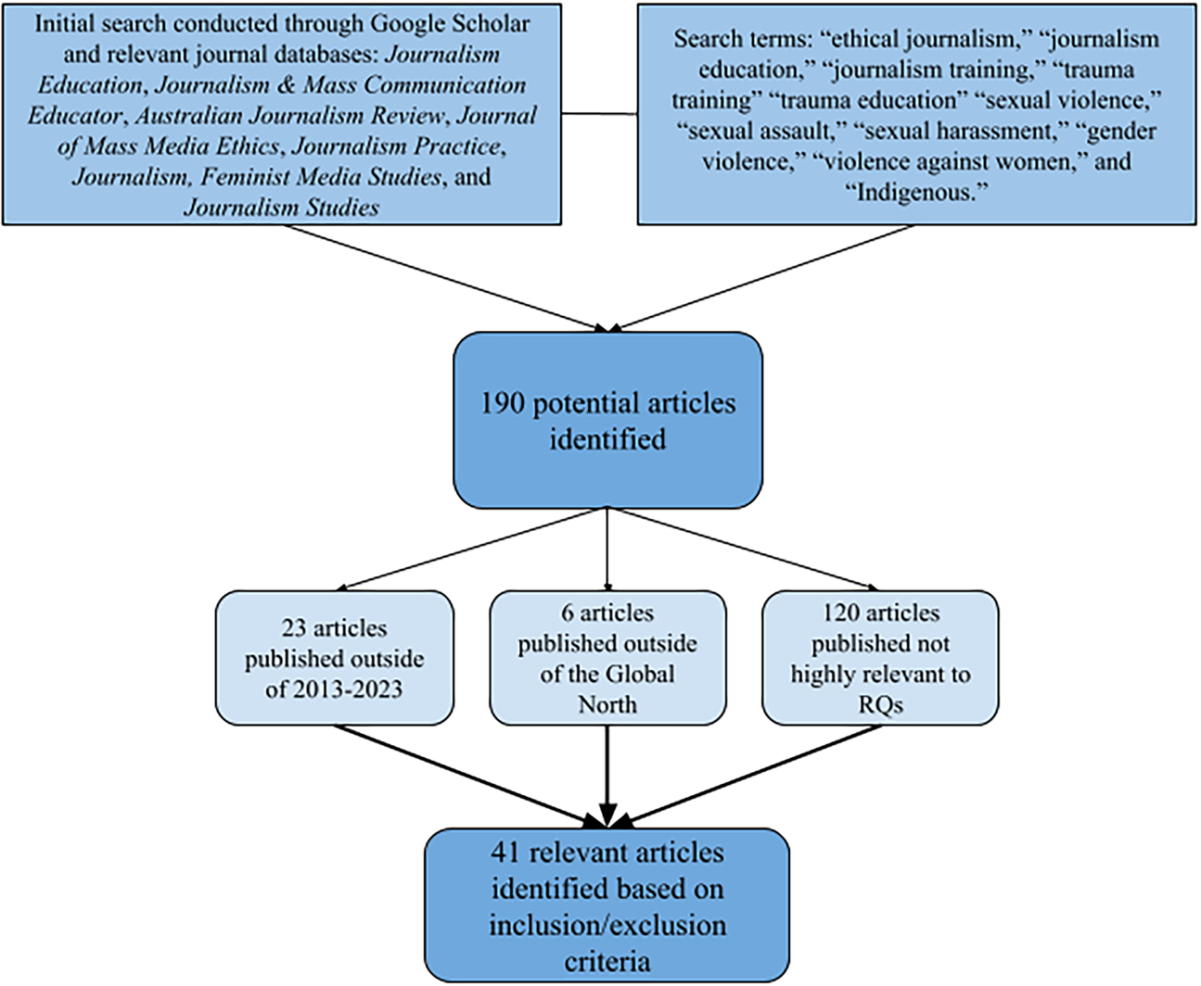
Literature search flow diagram.

We conducted a thematic literature review inspired by Brewer (2016). Brewer described using “Punch's (2009) process, which involves the following steps: summarizing, documenting, organizing, analyzing, and synthesizing the literature” (as cited in Brewer, 2016, p. 134). We followed the same approach which allowed us to thematically understand the key issues, challenges, and gaps. Using key search terms, literature relevant to the research questions was identified, recorded, and coded using descriptive codes to develop themes (Braun & Clarke, 2006; Saldaña, 2011) (Figure 1).

The first search was conducted by the first author in 2023, yielding 190 potentially relevant articles that were inputted into a master spreadsheet for the review stage. After filtering the articles based on the defined literature parameters, including thematic relevance, geographic location (Global North), and publishing date (2013–2023), the authors identified 41 relevant articles. The articles used various methodological approaches such as qualitative, quantitative, and content analysis. Articles pre-2013 were only considered if they were highly relevant to our research questions and/or if they were highly cited using foundational concepts in the fields of journalism, journalism education, or sexual violence research. Four articles fit this criterion: Amend et al. (2012), Franiuk et al. (2008a), Franiuk et al. (2008b), and Sampert (2010). The authors collaborated throughout the review process with several discussions and analyses of the spreadsheet to ensure that the narrowed list of articles best fit the research questions.

### Critical Feminist Perspective

Using a critical feminist perspective, the review revealed how “gendered assumptions and hegemonic power relations are discursively produced” (Lazar, 2007). As such, we examined the interlocking forces of patriarchy, racism, colonialism, and misogyny that affect journalism (Collins, 1998; Easteal et al., 2015). Employing a critical feminist approach reconsiders common binary representations of victim-perpetrator, object-subject, or consumer-producer (Anderson-Nathe et al., 2013) that enable the continuation of violence (Lykke, 2016). We rejected these binaries and oversimplifications of blaming journalists and victim-blaming in media reports. Acknowledging power and oppression within journalism reporting on sexual violence contributes to our feminist intersectional lens (Crenshaw, 1991). While rates of sexual violence are often uneven across populations (Black et al., 2014), all people can experience this harm. Sexism, alongside several other forms of oppression such as racism, homophobia, and transphobia, impact the burden of trauma created and sustained by sexual violence (McAllister & Vennum, 2022).

### Theme Development

We assigned descriptive codes to the 41 articles in the spreadsheet under the heading “keywords” (Braun & Clarke, 2006; Saldaña, 2011) (Table 1).

**Table 1.** Spreadsheet Headings Used for Theme Development.

Article	Year of publication	Geographical context	Keywords	Argument	Recommendations	Our analysis	Quotes

Based on the descriptive codes and other data in the spreadsheet, we used an inductive approach (Braun & Clarke, 2006) to organize these codes into themes with our research questions in mind, but without a “pre-existing coding frame” (p. 83). Braun and Clarke (2006) describe a theme as representing “some level of patterned response or meaning” (p. 82). They note that the researcher determines the importance of a theme based on “whether it captures something important in relation to the overall research question” (p. 82). We identified two categories or clusters of themes: media content and media process. We sorted the seven themes to synthesize and connect the data as described in Table 2. During theme development, it became clear that certain articles were highly relevant to multiple themes or subthemes. Some subthemes also only contained findings from one or two articles; because these subthemes were highly relevant to our research questions, this was not a factor for exclusion, and we did not impose a minimum number of articles needed for a subtheme (Braun & Clarke, 2006).

**Table 2.** Themes and Literature Identified.

Themes relating to media reporting content	Number of related articles (out of 41)	Percentage of related articles	Literature
1. Prevalence of rape myths and rape culture (sub-themes: campus culture, rape myth acceptance, and complication of consent)	11	26.8%	Acquaviva et al. (2021); Eckert et al. (2022); Elmore et al. (2021); Hindes and Fileborn (2019); Franiuk et al. (2008a); Franiuk et al. (2008b); O’Boyle and Li (2019); Pitchford et al. (2021); Sacks et al. (2018); Sampert (2010); Siefkes-Andrew and Alexopoulos (2019)
2. Language of blame (sub-themes: linguistics and labelling and sensationalist language)	7	17.1%	Ali et al. (2020); Aroustamian (2020); Lussos and Fernandez (2018); Northcutt Bohmert et al. (2019); Royal (2019); Siefkes-Andrew and Alexopoulos (2019); Sutherland et al. (2016)
3. Problematic media framing (sub-theme: episodic and thematic framing)	7	17.1%	Aroustamian (2020); Baker et al. (2020); Cuklanz (2020); Easteal et al. (2021); Easteal et al. (2015); Northcutt Bohmert et al. (2019); Starkey et al. (2019)
4. Ignored intersectionality (sub-themes: racism, colonialism, and queer objectification)	9	22.0%	Baker et al. (2020); Cobb and Horeck (2018); Cripps (2021); Evans (2018); Jackson (2013); Jaffe et al. (2021); Lykke (2016); Morrison et al. (2021); Su et al. (2022),
5. Biased use of sources	5	12.2%	Cuklanz (2020); Easteal et al. (2021); Evans (2018); Johnston et al. (2015); Waterhouse-Watson (2016)
6. Structural challenges for journalists (sub themes: issue dualism, work precarity, and newsroom culture)	7	17.1%	Amend et al. (2012); Blumell and Huemmer (2019); Braganza (2020); Cohen et al. (2019); Franiuk et al. (2008b); Heckman (2023); Wilkinson and Winseck (2019)
7. Lack of education, training, and practical engagement with ethical guidelines	6	14.6%	Amend et al. (2012); Barnes (2015); Easteal et al. (2021); Seely (2019); Sutherland et al. (2016); Wake et al. (2023)

Due to the specificity and level of detail of the seven themes, we incorporated the discussion with the findings as described below.

## Media Reporting Content

As the first cluster of themes, media reporting content refers to the specific subject matter included in journalism on sexual violence. This cluster addresses our main research question by examining the ways news media reports on contemporary sexual violence, both pre- and post- 2017 #MeToo movement in the Global North. We found that despite the increased awareness of sexual violence that accompanied #MeToo, the literature indicated harmful reporting practices persisted.

### Prevalence of Rape Myths and Rape Culture

Although sexual violence and its impacts have garnished mainstream recognition, particularly since #MeToo, this literature review highlights the continued prevalence of rape myths and rape culture in media reporting (Eckert et al., 2022). Drawing from Benedict's (1993) widely cited conceptualization, rape myths are false, deeply rooted beliefs that blame survivors and excuse perpetrators. The articles in our review explain how press coverage of sexual violence can be misleading and harmful. Rape myths and culture can include negative stereotypes and false beliefs about sexual violence, survivors, and perpetrators (Du Mont & Parnis, 1999); spread through various mediums; and apply to multiple forms of violence (Franiuk et al., 2008a; 2008b). Rape myths are “both a symptom of and contributor” to the high occurrence of sexual violence in the Global North (Elmore et al., 2021, p. 530). We identified three sub-themes that call attention to these issues: campus culture, rape myth acceptance, and the complication of consent.

#### Campus Culture

University reputations often take priority over supporting survivors in media coverage of sexual violence. O’Boyle and Li (2019) analyzed U.S. newspaper coverage of campus assaults and found that both liberal and conservative media placed individual blame on survivors. Similarly, Siefkes-Andrew and Alexopoulo’s (2019) research on sexual assault coverage on US college campuses found that 40% of the 71 stories from their study included language casting doubt on survivors’ experiences. These findings show how campus culture reinforces victim-blaming trends.

Pitchford et al. (2021) analyzed media portrayals of sexual violence involving professors, finding that these cases are often “framed as private rather than *systemic* problems … obscuring the institutional power dynamics” (p. 375). Violence can also be embedded in the structure of academic higher educational institutions (Ahmed, 2012); yet, universities are often reluctant to critically examine their role in perpetuating rape culture (O’Boyle & Li, 2019). These studies indicate systemic power and privilege are still engrained in campus culture and influence journalism practices.

#### Rape Myth Acceptance

Our review found that rape myths persist in media reporting. Analyzing six English Canadian newspapers from 2002, Sampert (2010) found that news media upholds myths about uncontrollable lust and victim blaming. More recent research suggests this trend continues. Through interviews with 34 students and alumni from a US university, Acquaviva et al. (2021) found participants relied on media for information, which can be problematic because “rape culture-supportive content is prominent in the media” (p. 24). Additionally, Elmore et al. (2021)'s quantitative survey of 286 community college students examined connections between media portrayals and rape myth acceptance and found that “25% of participants … accepted the myth that ‘she asked for it,’ … and 40% of participants … accepted the myth that ‘he didn’t mean to rape’” (p. 538). Although Elmore et al. (2021)'s methodological approach took a limited binary (male/female) approach to gender, together, these articles suggest that rape myths remain accepted and perpetrated, even post #MeToo.

#### Complication of Consent

Emphasizing gray areas of consent is often present in media reporting, complicating it in harmful ways. In the post #MeToo context, Hindes and Fileborn (2019) examined Australian media reporting of the Aziz Ansari case, arguing that the gray area of consent is a new topic of media discourse. They found that most reporting lacked nuance in approaching coercion and consent, reinforcing gendered stereotypes portraying women as “gatekeepers” and men as “naturally aggressive pursuers of sex” (p. 639). By downplaying pressure, coercion, and power, media excludes violence from discussions (Hindes & Fileborn, 2019). Survivors become responsible for the violence inflicted upon them because of the manufactured ambiguity surrounding consent. The lack of nuance and clear descriptions of consent described by these studies in reporting reduces its importance, which upholds rape culture.

### Language of Blame

For decades, researchers have looked at how language and grammar choices in media reports about sexual violence impact how the audience perceives blame (Bohner, 2001; Clark, 1992; Henley et al., 1995). The subtleties of passive voice carry enormous power, causing readers to perceive survivors as less harmed and more to blame for sexual violence (Henley et al., 1995). Authors of more recent publications have similarly investigated the role of language through two sub-themes: linguistics as well as labelling and sensationalist language.

#### Linguistics

Siefkes-Andrew and Alexopoulos (2019) analyzed how active and passive voice can be used by journalists to attribute blame to the perpetrator or the survivor, and they found that 40% of stories included verbs that conveyed doubt towards the survivor's story (such as “claimed” and “admitted” versus “stated” or “reported”). Northcutt Bohmert et al. (2019) found suspect mitigation was created in media stories because passive voice “allows for crimes to occur, seemingly by no one, allowing for suspect responsibility to be substantially lessened” (p. 883). In their study of mishandling of sexual violence media reports on US campuses post #MeToo, Lussos and Fernandez (2018) found that journalists continued to use passive voice, leading to an increase in victim blaming and reduced responsibility of the perpetrator. These studies show that the linguistic choice of passive voice continues to shift blame away from perpetrators and attributes blame to those already subjected to violence.

#### Labelling and Sensationalist Language

Another rhetorical device is the problematic ways that survivors and perpetrators are named or labelled. Aroustamian (2020) described the consequences of labelling the survivor as an accuser: “at the very least, labelling a victim/survivor as an ‘accuser’ brings doubt. At its worst, it connotes an ulterior motive, mental illness, or even evil intent” (p. 8). Labels such as “accuser” focus the readers’ attention on the survivor's actions rather than the perpetrator's (Royal, 2019).

Ali et al. (2020) found news media uses words like “monster” or “beast” to describe perpetrators, depicting perpetrators as frightening strangers rather than people who often know the survivor. Royal (2019) described how extreme language continues to be used post #MeToo; labels such as “sleazy tyrant” served to differentiate Weinstein and created the impression that women are safer from sexual violence than they are. Royal (2019) also found that labelling perpetrators as “good” people shifts blame and responsibility away from perpetrators.

Linked to labelling is sensationalist language. Sutherland et al. (2016) explained that sensationalist language in media representations of gendered violence “can be used for various effects: to titillate, shock, fascinate, amuse or entertain, as well as to obfuscate or undermine the true nature of the crime” (p. 7). Sensationalism creates a false view that sexual violence is only perpetrated by strange, visibly dangerous people rather than known, close contacts. Consequently, the literature reveals that the media continues to use language that shifts blame onto survivors.

### Problematic Media Framing

Frames of blame attribution are subtle and pervasive and can be used by well-intentioned journalists. Media frames are defined as “cognitive schemas audience members use to process information [and] to simplify and neatly package the causes, consequences, and responsibility related to crimes” (Northcutt Bohmert et al., 2019, pp. 874–875). Northcutt Bohmert et al. (2019) found that in sexual assault cases, survivors are blamed more than any other victims in media crime reports. Cuklanz (2020) found that while press coverage of #MeToo news articles showed less overt victim-blaming compared to pre-#MeToo coverage, journalists often neglected structural power imbalances. Cuklanz (2020) argued that more coverage of sexual violence does not always improve it; instead, it can entrench rape myths and deter survivors from reporting. The subtheme in this section explores episodic and thematic framing pre- and post- #MeToo.

#### Episodic and Thematic Framing

The most prevalent framing of sexual violence was episodic/individual. In a pre #MeToo (2017) study, Easteal et al. (2015) described how language and framing were essential elements in media portrayals of gendered violence. They elaborated on Iyengar's (1996) theory to specify thematic framing, “which tends to contextualize issues and emphasize social responsibility, and ‘episodic’ framing, which tends to emphasize individual circumstances and responsibility” (as cited in Easteal et al., 2015, p. 105). Through a qualitative content analysis, Aroustamian (2020) found small improvements in how the media used thematic framing over episodic framing post #MeToo in high-profile cases in the US. Aroustamian (2020) attributed these positive changes to increased visibility, social media advocacy, and the presence of anti-sexual violence organizations.

In contrast, thematic (structural/contextual) frames were much less prevalent framing within our review. In their study of sexual violence media reporting in the music industry in Australia, the US, and the UK post #MeToo, Baker et al. (2020) described how journalists were moving towards thematic frames that contextualized sexual violence in the music industry. However, through content analysis, Baker et al. (2020) found that journalists are still framing the stories within a context that privileges white females as survivors and largely ignores racialized and 2SLGBTQI + people. Starkey et al. (2019) emphasized that media frames are also culturally dependent. Considering the power of media representation, the literature showed that contextual framing is needed rather than episodic or individualized frames because of their influence on media consumers and ability to reaffirm victim blaming.

### Ignored Intersectionality

In contemporary journalism, there is a disregard for people with multiple, overlapping identities and how positionality impacts sexual violence and media reporting (Cripps, 2021). The #MeToo (2017) movement has “largely benefitted white, middle and upper class cis-hetero women as it simultaneously has elided the complexities of race, sexuality, class, and nation-status” (Su et al., 2022, p. 2). This exclusion renders only one type of survivor visible and upholds other forms of oppression. Intersectionality describes a lens for understanding how people's multiple identities impact their relationship with systemic privilege and oppression (Crenshaw, 1991). Three subthemes guide this discussion: racism, colonialism, and queer objectification.

#### Racism

Race has largely been ignored in media reporting on sexual violence, including prior to and during the #MeToo movement. Jackson (2013) examined the Megan Williams sexual assault case, where she, a Black woman, was kidnapped, tortured, and raped by six white men and women. Racist framing is used by the news media against both racialized men as “natural perpetrators” and racialized women as “un-rape-able” (Jackson, 2013, p. 50; see also Benedict, 1993). Jackson (2013) found that all the journalistic coverage missed the broader social implications of reporting on the white female perpetrators; sexual violence is about power, not sex. Anti-Black racism was particularly pervasive in this case, which aligns with Lykke's (2016) study that found Black women as largely absent from the discussion about sexual violence. Lykke (2016) conducted a content analysis of magazine articles and examined how the media use visibility and denial as mechanisms to reinforce racism and sexism. Similarly, Evans (2018) discovered that white women were most reported as survivors in the New York Times, and white “sources comprised 70.3% of the total group [of the 182 individual sources], easily the most often used” (p. 4). These studies provide important analyses surrounding race, gender, and misogynoir to demonstrate how racism functions in news reporting on sexual violence.

#### Colonialism

Colonialism is another factor that negatively impacts news media reporting of sexual violence. Notably, Khan et al. (2023) identified how sexual violence is one of the many ongoing, oppressive tools of settler colonialism. Reflecting on their analysis of print and online news media from 2011 to 2018 in Canada and Australia, Cripps (2021) used Indigenous research methodologies to uncover how stereotypes about Indigenous women were upheld in media reporting on sexual violence. While Cripps (2021) emphasized stereotypes about Indigenous women revolving around “goodness,” “rescuability,” and “assimilation,” there were also concurring stereotypes around “promiscuity” and “wantonness”. Indigenous women do not fit the myth of an ideal, worthy victim, presenting their experiences as “less newsworthy” (Cripps, 2021, p. 304).

In articles recounting the horrific experiences of Lynette Daley and Cindy Gladue, Cripps (2021) identified dehumanizing, sensationalist, victim-blaming, and racially stereotyped language. With accounts as part of the public record, this further perpetuates “colonial discourse that has denied Indigenous women their stories” (p. 316). Media framing of sexual violence survivors perpetuates victim blaming and erasure of dignity for those not of the dominant racial and social class. This harm is a part of the continuation of colonialism, racism, and sexism (Cripps, 2021).

#### Queer Objectification

Morrison et al. (2021) found that compared to stories involving straight, cisgender survivors, stories about LGBTQ survivors were more likely to include dehumanizing frames, sensationalist and body-objectifying elements, a lack of privacy, more victim-blaming, and increased stereotyping. This suggests that news reporting in Canada on sexual violence against 2SLGBTQI + peoples may often be dehumanizing and sensationalist. While sexual violence and media reporting practices contain gaps, this issue particularly impacts people in imbalanced ways (Jaffe et al., 2021). Moreover, while Baker et al.'s (2020) research found that gender inequality in the music industry was addressed more positively after #MeToo, media coverage still focused on white, middle-class women and largely ignored the stories of Indigenous, racialized, and 2SLGBTQI + peoples.

The findings in this theme are consistent with broader research on heightened violence, transphobia, and cissexism (Chan et al., 2023) as well as racialized queer erasure (Meyer, 2019) and colonial violence (Hume & Walby, 2021) in news media. Post #MeToo, reporting practices still employ racist, sexist, colonial, and cisheteronormative practices to erase experiences (Cobb & Horeck, 2018). Therefore, intersectionality and the multiple, coinciding forms of oppression is an important theme to consider in news reporting practices on sexual violence.

### Biased Use of Sources

Who is quoted and allowed to speak is critical in how media consumers perceive stories. Johnston et al. (2015) found that “journalists cultivate a select group of ‘official’ sources” to cite, which overlooks more expansive or opposing views routinely (p. 249). This structural bias not only excludes survivors’ voices but also discredits survivors as experts in their own experiences.

Waterhouse-Watson (2016) argued that including authoritative sources like police reinforces the myth that women lie and authority figures are more “trustworthy.” Evans (2018) noted that journalists disproportionately featured white female survivors despite racialized and lower socio-economic groups being over-represented in sexual violence cases. Similarly, Cuklanz (2020) described feminist and non-male voices as “largely absent from mainstream news coverage” and recommended including quotes by survivors or their representatives (p. 256). Easteal et al. (2021) agreed, recommending survivors and advocates as sources, while also suggesting media training for community spokespeople. These studies showed that excluding survivors or advocates as sources entrenches rape myths, empowers perpetrators, and silences survivors.

## Media Reporting Process

### Structural Challenges for Journalists and Journalism

Through our review of the literature, studies also uncovered structural obstacles to improving reporting practices for stories of sexual violence. This theme specifically addresses our research question on the barriers to ethical reporting on sexual violence that journalists face. These challenges include three subthemes: issue dualism, work precarity, and newsroom culture.

#### Issue Dualism

Franiuk et al. (2008b) conducted a case study about rape myths in traditional media by examining the Kobe Bryant sexual violence case and determined that journalists want to appear neutral, resulting in more skepticism for the survivor than the perpetrator. “Issue dualism” is when journalists aim to balance both sides of an issue, but this “problematically stresses the opinions of a few” (Blumell & Huemmer, 2019, p. 939). Blumell and Huemmer (2019) ask journalists to consider how a quest for perfect objectivity might fail to account for societal power imbalances. Similarly, Callison and Young (2020) call for a “decolonizing of ‘fairness and balance’” (p. 23) in their critique of objectivity because journalism fails to account for “intersectionality and Indigenous feminisms” (p. 7). Thus, the literature reveals that the quest for objectivity can contribute to the marginalization of those in less powerful positions.

#### Work Precarity

Journalists face increasingly precarious working conditions, making writing stories that follow ethical guidelines more challenging. Wilkinson and Winseck (2019) describe the precarity of journalists’ employment in Canada, including “plummeting revenues, falling profits, waning audiences, budget cuts, newspaper closures, scaled back production schedules, and news workers laid off” (p. 374). More in-depth journalism using contextual frames is expected with less time, less job security, and little to no training (Amend et al., 2012; Braganza, 2020). Yet, even considering these conditions, the onus is still placed on journalists to “fix the problem” of media stories that perpetuate rape myths. As a consequence of work precarity and these difficult conditions, Cohen et al. (2019) found that many journalists report feelings of demoralization, a sense of failure, and a “disappointment in not being able to produce meaningful content that contributes to public knowledge” (p. 18). The literature demonstrates that work precarity negatively impacts journalists and their ability to follow ethical guidelines on sexual violence reporting.

#### Newsroom Culture

The culture of newsrooms in the Global North is demanding and often lacks diversity, which poses challenges for many journalists. Through a focus group with seven Canadian journalists, participants described intense pressures such as “deadlines, tracking down sources for stories, and demands from the editorial desk” (Amend et al., 2012, p. 239). They also described a competitive work environment not conducive to producing diligently ethical stories that take time to write. Heckman (2023) described traditional newsrooms in the US as overwhelmingly white and “macho.” Callison and Young (2020) also emphasized the “lack of diversity and whiteness in newsroom [as] untenable and indefensible” (p. 6). They explained that legacy media in North America has been part of the structure of upholding structural racism and sexism. These studies exemplify that structural challenges can impact journalists’ ability to produce ethically framed and survivor-centric stories on sexual violence.

### Education, Training, and Engagement with Ethical Guidelines

In this final theme, we highlighted the lack of formal education and training not only in trauma literacy but also in engaging with ethical guidelines around sexual violence media reporting. Amend et al. (2012) examined journalism education programs in Canada and found that most journalists have not been trained or are not equipped to deal with ethical dilemmas or vicarious trauma. Amend et al. (2012) concluded that inadequate training in journalism programs and in the workplace can lead to retraumatization of survivors, ethical violations, and a lack of social change. Similarly, in their study in Australia and New Zealand, Wake et al. (2023) found that nearly all journalist educators agreed that it is necessary to build trauma resilience. They reported barriers like “lack of specialist knowledge and training materials, accreditation and budget issues, and lack of scope within existing unit and course learning outcome objectives” (p. 117). Most felt that their graduates left without knowing how to interview a trauma survivor without inadvertently causing more harm. In Wake et al.'s (2023) study, educators noted that racialized and marginalized people are often under-represented in journalism faculties.

Barnes (2015) examined how journalists are trained to deal with trauma, and whether this must be done in student education programs or the workplace. Barnes found that educators in Australia and New Zealand were confused “about what and how to teach trauma awareness” (p. 128) and suggested a critical need for engagement with guidelines. Sutherland et al. (2016) found there was little research into how the media used reporting guidelines about sexual violence and if organizations and journalists were aware of them. Seely (2019) described how many journalists in the US reported a lack of trauma literacy or a culture of discussing feelings or experiences in their workplaces; journalists they interviewed detailed negative mental health effects, including anxiety, fear, and guilt. Without adequate training in trauma literacy, the literature shows that journalists are not empowered to create ethical stories about sexual violence and struggle to balance their own needs within a rigid newsroom culture.

Easteal et al. (2021) compared the content and framing of journalists’ news stories before and after a gender-based violence workshop to assess the effectiveness of such interventions. In the workshop, journalists learned about principles that should be followed to properly contextualize stories on gender-based violence. The authors noted some improvements in the workshop participants’ media story framing, such as including resources, using various sources, and including social context (Easteal et al., 2021). From these studies, we see that within journalism education and on-the-job training, additional time, skills, and engagement with ethical guidelines is necessary for journalists who report on trauma and sexual violence. Considering these structural constraints on journalists, academic researchers must also include this context when designing, conducting, and writing studies on journalism and sexual violence reporting practices.

## Conclusion

Through this review, academic research has identified a critical gap in news media reporting: even post #MeToo, journalist practices can still problematically portray sexual violence, exacerbating harmful and lasting impacts for survivors. The literature suggests that despite increased coverage of sexual violence after #MeToo and the prevalence of guidelines for reporting, more needs to be done to ensure media do not perpetuate rape myths.

The literature we examined demonstrated how the quest for objectivity in journalism can be at odds with the feminist framework of believing survivors (Blumell & Huemmer, 2019). Media can also be gatekeepers around whose stories are told and are considered “newsworthy” (Cripps, 2021), which is important since the #MeToo media movement largely reflected the experiences of cisgender, heterosexual, white women and left Indigenous, Black, Brown, people of colour, and 2SLGBTQI + people out of the conversation (Cobb & Horeck, 2018; Cripps, 2021; Evans, 2018; Jaffe et al., 2021; Morrison et al., 2021). In a similar way, academic research also privileges these specific identities. For example, many empirical research articles that we examined used a binary approach to gender, thus systematically excluding people who do not fit into this normative, oppressive organization structure. This issue underscores what is missing in the existing literature and future researchers must account for less rigid gender categories when designing studies and in data collection.

Based on these findings in existing research, additional studies on continued education and on-the-job training will be essential to understand and support journalists to address critical issues like sexual violence reporting and trauma. Morrison et al. (2021) recommend local partnerships where journalists can learn from community organizers, activists, and survivors. We suggest extending these collaborations with academics and researchers for the purposes of knowledge mobilization and exchange within educational settings. Future research should gather data from journalists through interviews, focus groups, and surveys to better understand training and reporting practices, enabling more informed recommendations for ethical reporting. These investigative practices should include an in-depth evaluation of the constraints that journalists face and how they can be supported. Collaboration between academics, journalists, community groups, and survivors through ongoing research has the potential to challenge rape myths, victim blaming, and white male supremacy.

Sexual violence has devastating impacts, and the themes we described are not only about individual journalists or empirical studies but how these issues fit into the wider reality of sociocultural powers that accept and perpetuate the continuum of violence (Kelly, 1988). The research in this literature review demonstrates that gaps in media reporting still exist; however, these gaps are often not understood within the larger context of how structural violence influences and manifests through sexual violence. While we currently see and foresee additional challenges related to academic freedom and funding in higher education as influenced by the North American and global sociopolitical context, the approach to change must exist on multiple fronts within and beyond academia. Academia plays an important role in examining current practices and constraints; however, it is unrealistic for individual journalists or researchers to bear this responsibility alone. Rather, collective action must prioritize systems rooted in justice, liberation, and safety. As such, our shared futures and interconnectedness as human beings depend on our ability to act upon and against systems of oppression that manifest through sexual violence.
